# Barriers and Facilitators to Participation and Key Components of Sleep Health Programs: Perspectives for the Corporate Work Environment

**DOI:** 10.1097/JOM.0000000000002991

**Published:** 2023-10-18

**Authors:** Paula R. Pienaar, Astrid R. Bosma, Dale E. Rae, Laura C. Roden, Willem van Mechelen, Estelle V. Lambert, Cécile R.L. Boot

**Affiliations:** From the Health through Physical Activity Lifestyle and Sport Research Centre and Division of Physiological Sciences Department of Human Biology, Faculty of Health Sciences, University of Cape Town, Cape Town, South Africa (P.R.P., D.E.R., L.C.R., W.v.M., E.V.L.); Amsterdam UMC, Vrije Universiteit Amsterdam, Department of Public and Occupational Health and Amsterdam Public Health Research Institute, Amsterdam, the Netherlands (P.R.P., A.R.B., W.v.M., C.R.L.B.); School of Life Sciences, Faculty of Health and Life Sciences, Coventry University, Coventry, United Kingdom (L.C.R.); Human Movement and Nutrition Sciences, Faculty of Health and Behavioural Sciences, University of Queensland, Brisbane, Australia (W.v.M.); and School of Public Health, Physiotherapy and Population Sciences, University College Dublin, Dublin, Ireland (W.v.M.).

**Keywords:** health promotion programs, sleep health, workplace, employee perspectives, qualitative research

## Abstract

**Objective:**

The aim of the study is to explore the barriers and facilitators of participation and key components for sleep health programs designed for corporate work environments.

**Methods:**

Semistructured interviews with corporate executives and occupational medicine specialists in the decision making and management of workplace health promotion programs (WHPP) within their companies were held before and during COVID-19. Interviews were transcribed verbatim and analyzed using thematic content analysis to identify themes.

**Results:**

Barrier and facilitator themes emerging from the data include sleep health awareness, work culture, work-family balance, and confidentiality. Key components for sleep health programs included the following: identifying the need for a program, incorporating sleep health risk screening to WHPP, and promoting sleep health by raising awareness thereof.

**Conclusions:**

The identified barriers and facilitators to employee participation and key components of an ideal sleep health program provide guidance for further WHPP.

## Abstract 2

Sleep is essential for health and work performance yet may be compromised by demanding corporate work demands. The sleep health of corporate executives can be supported by identifying barriers and facilitators to participation in sleep health programmes and considering key components for its development in the corporate work environment.

## Graphical abstract

**Figure:**
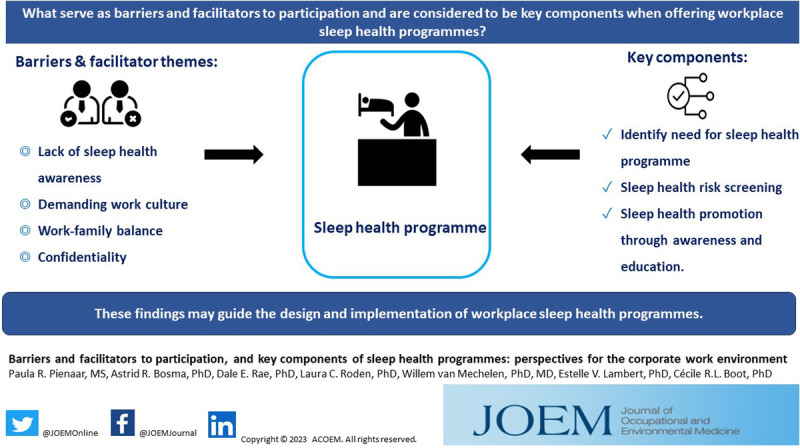

**LEARNING OUTCOMES**

After reading our study, the reader will be able to:

1. Distinguish how employees in corporate executive positions would benefit from optimizing their sleep health and value that their influence has the potential to promote the inclusion of sleep into workplace health promotion programs within their organization.
2. Describe and recognize barrier and facilitator themes such as sleep health awareness, work culture, work-family balance, and confidentiality for consideration when aiming to encourage participation in sleep health programs within a corporate work environment.
3. Identify potential key components that could facilitate the development and implementation of a sleep health program designed for the corporate work environment.

While the negative consequences of poor sleep health (insufficient sleep duration and poor sleep quality) on physical and mental health have been well described,^1,2^ more recently, the effects on workplace performance have come to light. In addition to the medical^3,4^ and psychological consequences^5^ of chronic poor sleep health, the social and occupational functioning of working adults can also be impacted. Studies from many countries have found that employees often work after a night of poor sleep.^6–8^ Poor sleep health has thus been shown to be particularly problematic to employees within managerial positions where it has been reported that 40.5% get less than 6 hours of sleep.^9^ This too may lead to a variety of cognitive deficits, including an inability to maintain attention, decreased alertness, delayed reaction time, altered emotional and information processing, and a general inability to think clearly.^10^ Moreover, it has been recognized that well-rested employees are less absent from work, perform better when at work, make better decisions, and interact more positively interpersonally.^11–13^ From an economics perspective, there is evidence suggesting that healthy employee sleep and corporate success are directly correlated, and consequently, it has been proposed that business leaders seek ways to translate this research into work policies to not only improve employee health but also to improve their own financial bottom lines.^14^ Considering the negative impact of poor sleep on the health and work performance of employees, and that it evidently comes at an economic cost to organizations, it is vital to provide effective support with solutions to help prioritize good sleep habits.

Unfortunately, the positive impacts of good sleep health on work performance are often underappreciated by employees in managerial or leadership roles. Pressure to meet challenging performance objectives drives these employees to extend work hours or to create unrealistic work schedules, often favoring perceived productivity gains of additional work time over productivity losses due to impaired sleep.^12^ As such, sleep health is not widely prioritized in organizational policies and practices.^15^ The role of workplace culture has also been found to impact on employee sleep. There is evidence to show that a workplace culture consisting of high work demands, especially for employees at management level, may indirectly impact sleep via extended work hours and elevated job stress.^16^ Corporate executives are a particular demographic group engaged in a high-pressure work culture and with their level of responsibility and demanding work commitments. It is not surprising that they may struggle with time availability, often resorting to extended work hours at the expense of adequate sleep. Many companies have adopted Workplace Health Promotion (WHP) Programs targeting health-risk behaviors such as poor nutrition, physical inactivity, smoking, or alcohol consumption as a means to reduce risk for noncommunicable diseases or mental health disorders.^17,18^ Programs that promote sleep health, however, are still lacking or are limited, despite strong evidence for the relationship between insufficient sleep and adverse physical and mental health outcomes.^19,20^ In addition, the success of programs focusing on lifestyle behaviors are often limited by low participation rates and lack of adherence.^21,22^ In response to these challenges, and to enhance the sustainability of behavior change, previous research on the success of WHP programs indicated that employees should be involved in the planning and implementation processes.^23^ As such, the role of corporate executives is vital in contributing to the adoption and successful implementation of WHP programs within their organization, as they hold the decision-making power in approving such health strategies throughout their business. Furthermore, as business leaders, their involvement in the organization’s health promotion planning, policies, and practices mean that their knowledge and past experience hold value in developing and enhancing a successful sleep health program.

The importance of leadership to the success of wellness initiatives within organizations is widely acknowledged, but there remains relatively little research on the actual impact that their leadership may have in promoting employee sleep health. It has been demonstrated that employees regard leadership support based on the programs and policies they provide. Also that if companies invest in wellness initiatives, these initiatives are acknowledged and recognized by employees.^24,25^ As such, the actual participation of management, such as corporate executives, may demonstrate their commitment to employee health through ensuring that subsequent WHP programs are acted upon in the workplace. Interviews with managers who play an integrative role of program implementation can provide valuable insights into the barriers and facilitators to employee participation in WHP programs of organizations, along with what they would consider key components to include in such a program. The outcome of such insights may therefore provide a foundation for developing an ideal framework of sleep health programs, which ultimately can be adapted beyond the corporate setting into all segments of the business.

While these objectives may yield promising insight on sleep health management from organizational leaders, one cannot ignore the recent impact that the COVID-19 pandemic played for WHP programs and sleep health in particular. Many studies have shown that sleep patterns and behaviors changed among the general populace worldwide during lockdown,^26–28^ but studies considering specifically people in the corporate setting have not been carried out. Earlier studies on the effect of COVID-19 restrictions such as home confinement showed that these were linked to sleep problems in employees.^29^ While some studies showed an increase in sleep duration that may have been due to work flexibility and less commuting time,^30,31^ others reported sleep disruption mainly due to increased stress and anxiety.^32^ Incorporating sleep health within WHP programs during the period of COVID-19–related restrictions therefore brought new challenges compared with pre–COVID-19 times. By exploring insights from the key players in WHP program planning, we may gain a better understanding of perspectives on sleep health within a corporate setting.

This qualitative study aims (*i*) to explore perceived barriers and facilitators of participation in a sleep health program for corporate executives and (*ii*) to gain insight from corporate executives and occupational medicine specialists around key components of an ideal sleep health program that could be implemented as part of a WHP program. Because of the COVID-19 pandemic and associated lockdown that took place during data collection, the study was adapted to include a third aim: to reinterview participants after the first wave of the pandemic to determine whether insights and perspectives around a sleep health program had changed.

## METHODS

### Study Design and Setting

This study made use of a qualitative research design based on semistructured interviews with senior business managers and occupational medicine specialists. This specific setting was chosen because corporate wellness programs are exclusive to senior business managers who, together with occupational medicine specialists, are typically involved in the decision making and management of WHP programs within their companies. Accordingly, the present study used an illustrative case study design.

Initial interviews were conducted in-person before the first wave of the COVID-19 pandemic in South Africa (February–June 2020) and follow-up interviews took place remotely between the first, second, and third waves of the pandemic (December 2020–December 2021). The study was approved by the University of Cape Town’s Human Research Ethics Committee and all participants provided written informed consent upon agreeing to take part in this study.

### Participants and Recruitment

Participants were recruited from a larger national executive health database consisting of health risk assessment (HRA) data as previously described.^33^ To be eligible for this convenience sample, participants had to be full-time employees in senior or executive management positions and have participated in their workplace HRA. Moreover, occupational medicine specialists in the database had to be active in the service provision of such workplace HRAs and/or the implementation of WHP programs. Participants were excluded if they were shift workers. Email invitations describing the aims and background of the study were sent to participants who had their HRAs in 2018 or 2019. Those willing to participate were contacted via email to arrange a date and time most convenient for their interview. Recruitment was halted at the start of the pandemic, resulting in a total of seven participants (2 men and 5 women) of which five were corporate executives (senior business managers) and two were occupational medicine specialists. To allow for longitudinal comparisons, the same participants were invited via email for a follow-up interview in 2020. Given the social distancing restrictions at the time, the consenting participants (*n* = 7) were provided with the option to be interviewed telephonically or using Microsoft teams at a date and time most convenient for them.

### Data Collection

Where possible, two members of the research team were present during the interviews, which were audio-recorded using a digital sound recording device. The primary researcher (P.R.P.) facilitated the session by explaining the purpose of the study and by guiding the interview by using predetermined, open-ended questions designed to elicit conversation around potential barriers to and facilitators of participation in a sleep health program, perceptions regarding the need for a sleep health program among corporate employees, and views on key components in an ideal sleep health program. The questions used for the follow-up interviews were adaptations of the initial interview questions to allow for consideration of the impact of the COVID-19 pandemic on the previous responses, in relation to sleep health and WHP programs. The complete question schedule used in both interviews is shown in the Supplemental Digital Content (http://links.lww.com/JOM/B424). The follow-up interviews were conducted using the Microsoft Teams© application, and in all interviews, the researcher and participant had their laptop camera switched on for a virtual face-to-face session. Both initial and follow-up interviews lasted no longer than 60 minutes and were conducted in English.

### Data Analysis

Audio files were transcribed verbatim and thematic analysis was used according to Braun and Clarke^34^ to analyze the collected data. Two researchers then independently coded the transcripts line-by-line in Microsoft Word (P.R.P. coded all transcripts, A.B. coded 25% of the transcripts). The coding process was guided by interview topics, using a deductive approach. P.R.P. and A.B. compared and discussed initial codes until consensus was reached, to ensure consistency of the analytical process. Next, codes were explored for similarities and discrepancies, ultimately grouping and combining codes into themes. After extensive discussions within the research team, themes were mapped onto a “pre-“and “during” COVID thematic coding tree. Representative quotes from the interviews were added to illustrate the findings.

## RESULTS

Themes were identified within the two main topics of this study: (1) barriers to and facilitators of participating in a sleep health program and (2) key components of an ideal sleep health program designed to be implemented within the corporate work environment. An overview of the themes that emerged can be found in Table 1.

**TABLE 1:** Themes Regarding the Barriers to and Facilitators of Participation in a Sleep Health Program and the Key Components of an Ideal Sleep Health Program

Barriers to and facilitators of participating in a sleep health program
1. Lack of sleep health awareness
2. Demanding workplace culture
3. Work-life balance
4. Confidentiality
Key components of an ideal sleep health program
1. Identifying the need for a sleep health program
2. Health risk screening
3. Education and raising awareness

### Barriers to and Facilitators of Participating in a Sleep Health Program

#### Lack of Sleep Health Awareness

At the corporate level, the lack of awareness and guidance related to sleep health were considered barriers to participation in a sleep health program. While the importance of sleep for general health was recognized, participants admitted that organizations did not prioritize the development and implementation of sleep health programs, as they did not know how and where to begin. Furthermore, it was evident from the interviews that despite the general knowledge around sleep health, appreciation of its significance for work performance and cognitive benefits was lacking. To resolve this, interviewees thought about how to overcome this barrier by indicating that it was vital to convey the link between adequate sleep and how it can optimize work performance, particularly to business leaders, as it would facilitate participation and make the business case for sleep health programs.

Another barrier raised within the context of sleep health awareness was corporate executives denying having sleep problems or simply ignoring their sleep-related concerns, despite the recognition of experiencing exhaustion during the day at work. Participants felt that “lack of sleep” was standard practice among corporate employees and that although they could fall asleep at any opportunity, indicating their exhaustion, they denied needing any sleep support. It was further mentioned that participation in a sleep health program would be low if employees did not recognize or admit they battle with their sleep.

*“So it would be really beneficial, and I think what happens and in my experience and talking with people in corporate environments is that they become so accustomed to these sleep habits that it’s their new norm. And so, it becomes so much difficult to undo, they don’t actually realize that the long-term effect of it is extremely bad for them.” (*Pre–COVID-19*, SI07A)*

#### Demanding Workplace Culture

The workplace culture of organizations was perceived as a barrier to participation in a sleep health program. Participants expressed that the work demands set at the managerial level may hamper participation in a sleep health program. This barrier was relevant before and during the COVID-19 pandemic as it pertained to the stress-provoking and demanding management style adopted within the organization. Workplace culture was governed by expectations of having to be available after hours and to complete work deadlines at short notice, resulting in high work pressure with challenging working days and a lack of time to prioritize sleep or adopt effective sleep management strategies.

“*So, there may be expectations of ability, and reasonable availability, productivity driven you must be able to meet particular targets even if it’s a weekend, or a Sunday, or at night.” (*Pre–COVID-19*, SI01A)*

During COVID-19, these work expectations were further exacerbated because of the demanding expectations from clients, which were experienced by managers across the corporate environment.

“A*nd then also if you have very senior managers sending emails at all hours of the day, or night it kind of sets the tone for everyone else. So, yes there isn’t a verbal expectation, it’s not expressed that you need to be available all hours of the day, or night, or that’s how you should be working, but it’s kind of indirectly saying to everyone you know, that this is okay...*” (During COVID-19, SI05B)

In thinking about ways in which to overcome this barrier, some participants suggested that a change in work policies was needed to allow for more flexibility around work times. Given that they would be more likely to participate if a sleep health program, or parts thereof, were made available during work hours, work flexibility was considered as a facilitator to participation and would make it easier for employees to prioritize sleep health. Likewise, employees felt that they would participate in a sleep health program, provided that daily work meetings be reduced allowing for work to be completed during working hours, which in turn would allow more time for sleep support.

#### Work-Life Balance

According to participants before and during COVID-19, a proper balance between work hours and family time would be a prerequisite and facilitate participation in a sleep health program. In contrast, the lack of balance, with work taking over family time, was raised a barrier. This concern for work-life balance was expressed by emphasizing employees’ resistance to spending the limited available time after work hours on participation in a sleep health program. Instead, they would rather spend it on family and home responsibilities. This was particularly emphasized in an interview before COVID-19:

“*So, it’s difficult to get through the importance of it as a concept because you know, it’s just “Get on with it and sleep.” But it’s also about work-life balance, something we don’t have, and all that sort of thing*.” (Pre–COVID-19, SI06A)

“*… if it is more efficient, and they do all go on a program, and their sleep is improved perhaps more time at home, or family time….*” (During COVID-19, SI07B)

Before the COVID-19 pandemic, most employees were expected to work from their office and as such, would have to wake up earlier to be at work on time, and leave the office later to avoid traffic and long commute times. Given this, many would restrict their sleep duration during their workdays. In contrast, during the pandemic when employees could work from home, this hindering factor was resolved. However, despite having the potential opportunity to lengthen their sleep duration, working from home too was accompanied by challenges. Participants felt that they experienced higher work demands and more stress having to combine work time and family care in a day, resulting in a lack of time and energy to invest in a sleep health program.

As such, another work-related challenge during COVID-19 was related to the changes within the work environment during the pandemic (i.e., working from home). It was felt that the lack of in-person or face-to-face contact would make the implementation of a sleep health program unfeasible or less likely to be effective and would complicate the monitoring process and inhibit employee participation.

“*I think it’s harder to implement it remotely, so I think that is a barrier too, because employers have probably got less control over their employees’ routines when they are working remotely….”* (During COVID-19, SI03B)

Technology was also implicated in the interference of work-life balance and hindered participation in a workplace sleep health program. Before COVID-19, the use of technology was associated with a generational gap in that older employees were not keen to participate in a program if it were too digitalized.

“*It depends on the person I think sort of younger people probably like, who are more sort of on their phones would probably take in up better, but then I mean, I’ve got some older patients who I think you’d end up causing more stress by giving them a sleep app. It’s a generational thing I think.”* (Pre–COVID-19, SI03B)

During COVID-19, participants reported that organizations struggled with the use of, and access to, technology and online systems, which were required at the time. Therefore, several respondents felt that this could further complicate the implementation of and participation in a sleep health program that could be provided online. In addition, it was pointed out that it would be ineffective to provide online support, as it would not reach all employees, or fail to entice those who do not have adequative technological skills.

“*So yes, I was saying that I think for me what was the barrier was mainly, you know, the technology that we had to use at the time....”* (During COVID-19, SI02B)

A different view regarding online support was given by a few participants, however, whereby it could support work-life balance and facilitate participation. Specifically, participants felt that easy-to-use mobile apps and virtual interventions would provide a novel and welcoming angle on which to introduce an online approach to sleep health programs within the organization. As such, this option would allow employees to access a sleep health program at times most convenient to them, thus facilitating work-life balance.

“*I would think that you could, every second day get together on Zoom, and you would chat about your week, or couple of days that... it would be interesting first of all, to get them to mark their pattern of their sleep, and then you could sort of link up with them to find out how things had changed, then you’d convince them that maybe they needed a sleep thing....*” (During-COVID-19, SI07A)

#### Confidentiality

The issues of privacy and confidentiality were raised before and during COVID-19, as participants felt participation in a sleep health program would only be viable if full anonymity were guaranteed. Those who perceived lack of confidentiality as a barrier to participation were fearful that the organization’s wellness services may recognize and target them and that their jobs may be jeopardized as a result. More specifically, they were concerned that their acknowledgment of poor sleep and its effect on their job performance may expose them as being vulnerable, weak, and unable to cope with their work demands, ultimately threatening their credibility as business leaders. Another angle was given during COVID-19, where it was felt that online group meetings that discussed sleep health would deter employees from raising their sleep concerns due to the lack of anonymity.

“*Very, very senior managers saying like “I will not call in, because who’s going to see my file and who’s going to know?” So, we’re quite fine with calling in about a contract or some kind of legal issue, that’s OK, “I don’t mind giving you my ID number and you knowing who I am.” But when it gets to the more personal things, definitely not OK with accessing what’s available*.” (Pre–COVID-19, SI05A)

“*There is such insecurity around will it show my manager that I’m weak, and they don’t need me, and they can replace me, I’ve got to hold on to this job that you know, the job market is, so tough, and it just gets, so yeah, so confused, and they are not making good decisions, because they have not slept*.” (During COVID-19, SI04B)

### Key Components of an Ideal Sleep Health Program

#### Identifying the Need for a Sleep Health Program

Given the lack of awareness around the effects of sleep on health and workplace performance at the organizational level, the need for a sleep health program is not self-evident. Two different approaches to identify the need for a sleep health program were raised. First, the occupational medicine specialists in the group suggested doing a “needs assessment,” which involves the steps before WHP screening and implementation. Second, the corporate executives in the group proposed identifying the need for a sleep health program within the organization based on the lack of sleep health support for employees with sleep problems.

Occupational medicine specialists felt that through a needs assessment, the organization would emphasize the necessity for such a sleep health program, and by demonstrating the potential benefits, there would be a greater likelihood of influencing those in management to invest in sleep health.

“*So, there is a whole range of stuff in that primary preventive space it’s enabling, it’s shifting, it’s engaging, it’s motivating, so that’s essential that’s a groundwork that’s got to be there, and of course part of the needs in that, is to convey that there is a need, the degree of need, and set the scene for intervention.”* (Pre–COVID-19, SI01A)

Furthermore, corporate executives interviewed were adamant that employees needed a health program specific to sleep, as there was inadequate focus on sleep health, nor any process or support they could follow for their sleep concerns. Therefore, they anticipated that employees would value such a program if provided by their organization.

“*I think employees would just grab it [added by the authors: sleep health program] and run with it I can tell you that.”* (Pre–COVID-19, SI02A)

During COVID-19, occupational medicine specialists and corporate executives within the sample felt that a sleep health program was needed as no emphasis was given to sleep health. Instead, it was only addressed indirectly through topics on mental health and general fatigue.

“*So let me think about the corporate, where mental health and fatigue generally certainly have reached the general conversations at corporate, either by way of online talk sessions or by way of communications. Perhaps not as directly do they talk to sleep, let me say. Typically, it’s more around fatigue itself and stress and the demands of working from home, work-life balance difficulties. So sleep perhaps is implied there, but not perhaps as visible as it could be, as a specific component of the fatigue equation*.” (During COVID-19, SI01B)

“*I think it’s almost more important than it was before when we were having our normal way of life if you want to put it that way. I think we are desperate for better sleep, or good quality sleep now….”* (During COVID-19, SI05B)

#### Sleep Health Risk Screening

Both before and during COVID-19, participants felt that there was a requirement for identifying signs of daytime fatigue from lack of sleep, and symptoms of poor sleep, by a process of sleep health risk screening. Occupational medicine specialists gave their perspective from an organizational level, which involved large-scale screening procedures allowing for groups of employees to be stratified based on data collected about their sleep health status (e.g., sleep duration, sleep quality). The remaining participants reflected on sleep health risk screening at employee level with reference to identifying individual employees and providing them with the sleep health support they would need. However, in both instances, the shortage of sleep-specific screening to detect poor sleep health was made evident. During COVID-19, interviewees felt that sleep health risk screening and the appropriate management for those affected be mandatory or at least prioritized. The suggestion from some participants during follow-up interviews was that in response to sleep health risk screening, employees could have access to an online consultation regarding their sleep health risk.

“*… any sort of occupational health program there would be sort of a screening aspect to it, kind of you know, screening out people who are at risk you know. Do a risk assessment of the employees, and look at who, which employees are at risk of sleep problems, and then kind of, implement any interventions which might mitigate those problems.”* (Pre–COVID-19, SI03A)

“*So, I would enforce it to start with, so that people also get to understand that they shouldn’t be afraid of something like this, that it’s actually in their best interest and also support them in whatever are the outcomes you know. If it is that they need to get booked off for a week because they are depleted whatever it is, and that it should be understood that it’s part of remote working, it’s part of working in a pandemic, and yeah, I think I would just do something, that’s what I’m saying*.” (During COVID, SI03B)

In contrast to the responses that online screening and virtual sessions would facilitate the sleep health risk screening process, some respondents felt that employees would benefit more from a sleep health risk screening process if it included a face-to-face consultation with a healthcare provider. Collectively, the general feeling was that sleep health risk screening be conducted in conjunction with a feedback session. Irrespective of when interviews took place, participants felt that a workplace sleep health program would require an individualized approach to meet employees’ needs based on their health screening.

#### Raising Sleep Health Awareness and Education

At both interviews, before and during COVID-19, respondents felt that raising sleep health awareness within the organization, starting with the managers was critical. At the managerial level, there was indeed acknowledgment that corporate executives understood the importance of adequate sleep for general well-being, but how it could improve their work performance as business leaders was unclear to them. Furthermore, this could be extended into education, as participants explained that corporate executives needed education around strategies that would enable them to identify when their sleep is problematic so that they would know when to seek support and how to restore their sleep health. During the follow-up interviews, participants also spoke of educating employees on ways to manage sleep patterns and create structure to their days.

“*So, first of all we don’t actually know how big the impact is, so I’m not actually clear unless someone admits to me that they can’t sleep, or they don’t, sleep, or they’re tired, and other than you know observation … so it’s around an awareness program making people understand what it is, and then making people comfortable enough to say well yes maybe this does affect them, and then interventions to help them with that balance, so I mean that will actually be multipronged, it will be on education, and awareness.*” (During COVID-19, SI06B)

“*I think there’s always a need for sleep education and I think especially now when people’s routines are, so out of sync. They would definitely benefit from some education around sleep, and the importance of it.*” (During COVID-19, SI03B)

## DISCUSSION

This study explored the barriers to and facilitators of participation in a sleep health program and key components required for designing such a program, both before and during the COVID-19 pandemic. At both time points, the overarching themes emerging with respect to barriers and facilitators included sleep health awareness, work culture, work-life balance, and confidentiality. Specifically, the lack of sleep health awareness among employees was regarded a barrier to participation. Moreover, the demanding work culture, lack of work-life balance, and insecurities regarding confidentiality were perceived barriers that would discourage employees from participating in a sleep health program provided by their workplace.

When asked to conceptualize an ideal sleep health program, the following themes emerged: identifying the need for a sleep health program, incorporating sleep health screening into HRAs, and promoting sleep health awareness through education. There were no striking differences in the perspective of participants before and during COVID-19 as at both time points they felt that sleep health was not prioritized and consequently no sleep support systems were available for employees in need thereof.

A barrier for participating in a proposed sleep health program both before and during COVID-19 was the work pressure experienced in what was perceived to be a demanding workplace culture. The organization’s “corporate culture,” characterized by high work demands and time constraints, was considered as a factor that would hinder uptake and participation in a sleep health program. Because our participants provided input from a corporate setting of a predominantly male demographic, the given corporate culture is in line with similar groups described in the literature. For example, there is evidence that in male-dominated industries, this demanding work culture perpetuates a masculinized culture by promoting conformity to longer work hours. As such, the time constraints found to hinder participation in our study suggests that similar to previous studies, working overtime is normalized and an obligation to work additional hours “for the team,” often comes at the expense of healthy sleep habits.^35^ Another study exploring barriers to participating in WHPs found that time constraints that would limit participation were evident among employees who showed a preference to keep work and private life separate. A possible solution proposed in the literature was to enable participation in the educational aspects of a sleep health program during work hours, thereupon keeping the private life separate from work-related activities.^36^

The lack of work-life balance was another barrier that could discourage participation in sleep health programs. With new work structures adopted during COVID-19, additional strain was placed on work-life balance. This new way of working included working from home, which was mandatory during national lockdown and which posed new challenges that impacted on employees’ daily routines and priorities. As a result, working overtime left little time for any additional commitments, including participation in sleep health programs. Another important finding was the ongoing availability and communication between employees due to the use of online and digital technology during COVID-19. The constant connectivity was found to interfere with the boundaries between work and home time, leading to the encroachment of work into family time. This finding is in line with another study showing that during COVID-19, work-related technology use during home confinement and outside of working hours caused greater work-family conflict.^37^ Consequently, the new work environment with its altered daily routines and ongoing connectivity after work played vital roles in creating resistance to welcoming a sleep health program. The greater emphasis on work-life balance during COVID may indicate a sense of shifting priorities to value family over work, especially in times of distress. Nevertheless, as employees have recently returned to the office, or adopted a hybrid model for work, the restored work structure and schedules may provide a revived interest in protecting their sleep, thus increasing the likelihood of participating in a workplace sleep health program. To further explore this, additional studies would provide clearer insight.

The lack of awareness of how sleep is specifically linked to work performance was regarded as a barrier among corporate executives. This was because participants felt that if those at managerial level knew how optimizing sleep could improve their ability to perform more efficiently and effectively as business leaders, they would be more likely to participate and invest in sleep health within their organization. The ability to recognize and acknowledge one’s own poor sleep patterns was considered as a start to raising sleep health awareness among corporate executives themselves. In contrast, self-denial of suboptimal sleep health would make it difficult for organizations to introduce a sleep health program, as employees would not see the need to participate. This denial of having sleep health problems has been described previously to show that societal norms have encouraged the normalization of compromising sleep time for additional work time.^35^ Moreover, the barrier to participation due to lack of sleep health awareness may be different between corporate employees and employees in nonmanagerial roles. Managers may have easier access to knowledge on the importance of sleep on health outcomes, compared with employees without similar educational backgrounds or accessibility to health education. Nonetheless, it seems that managerial occupations are more likely to associate short sleep duration and irregular sleep patterns with work commitment and dedication. This too was reflected in a recent study exploring the perspectives of male employees on sleep, where working more and sleeping less were considered as a sign of machoism and stoicism for achieving greater work productivity.^35^ Despite the existing knowledge on general sleep health that managers may have, our findings indicated that organizations fail to dedicate efforts toward sleep health programs due to the lack of knowledge and experience of developing and implementing such projects.

The confidentiality barrier implicated among the present corporate executive participants were related to being fearful that they would feel exposed. Specifically, that by choosing to participate in a sleep health program, they may be considered to be less effective managers compared with their colleagues, thus risking their job. In a study of office employees, Klasen et al^38^ (2021) found that “privacy” was a perceived barrier to participating in a preventative strategy targeted at reducing long-term sickness absence. The psychological stigma attached to sickness absence was implicated as the reason for nonparticipation. This too may provide a plausible explanation within the present group as it is well-established that a bidirectional relationship exists between psychological well-being and sleep.^39^ The corporate executives for whom sleep may be problematic could feel marginalized, as having poor sleep may lead to the perception that they also require mental health support. Given this, overcoming the confidentiality barrier is an important step in improving participation. Providing employees with clear information on data security and privacy policies may help ease their fear of participating.

Our findings showed that to develop an ideal sleep health program, organizations are required to identify why it would be needed. This is in line with a statement made by Dawes^40^ (2001) emphasizing that to improve workplace health, companies should realize what is needed to create a baseline from which new workplace health schemes can be adopted. The National Institute of Clinical Excellence provides guidance on steps to conduct a WHP needs assessment and emphasizes the involvement of all stakeholders in identifying health priorities within the organization, and planning the health action necessary.^41^ Moreover, it is vital to conduct a needs assessment before program implementation as it would increase employee participation rates and avoid barriers that otherwise would be missed.^42^ Accordingly, after the needs assessment, organizations are guided to develop appropriate HRAs. Given this, it should be noted that for interviews with occupational medicine specialists, particularly before COVID-19, their perspectives on developing an ideal sleep health program were in the context of a WHP needs assessment at organizational level. However, when it was discussed by corporate executive participants, reference was made to assess the needs for a sleep health program based on the sleep health of employees at an individual level. Specifically, it was emphasized that currently, those with sleep problems had no support or workplace sleep health management plan, thus justifying the need thereof.

The WHP programs typically incorporate HRAs and biometric screening to stratify health risk based on the number of health risk factors present.^43^ As such, HRAs are tools that can be used for risk identification, risk assessment, and risk reduction.^42^ Sleep health risk screening could therefore be the foundation upon which the need for a sleep health program would be identified in the workplace setting. Subsequently, organizations can identify and tailor strategies to support employees requiring sleep health management. Taken together, the implementation of such programs begins with being aware of the importance of sleep health and identifying the need thereof which may be facilitated by sleep HRAs.

### Methodological Considerations

Having two successive interviews enhances credibility, as participants were able to reflect on their initial interview conducted before COVID-19. The longitudinal approach allowed for possible changes in the perspectives on sleep-related WHP programs resulting from the COVID-19 lockdown and altered work environment. Furthermore, the interviews were done in real time, in other words, in-person during initial interviews or online, using the camera for face-to-face communication during follow-up interviews. This made it possible to collect richer, more comprehensive information and context to responses, compared with gathering information through surveys or questionnaires. In addition, despite the COVID-19 restrictions that occurred during the study, all follow-up interviews were conducted online, which was advantageous as it allowed for easier accessibility and flexibility of the interviews and allowed participants to be interviewed in their own chosen space. In favor of this study was the focus on employees at management level and within the occupational health space. This provides a homogenous sample of corporate executives and occupational medicine specialists who came from a position of similar leadership responsibility and that may have the decision-power to facilitate or support a sleep health management component to their existing WHP. Given the lack of previous literature highlighting the insight of organizational leaders in this regard, the results of the present study may guide future situations so that a better management of the sleep health of corporate employees can be disseminated.

It is, however, important to acknowledge the small sample size within this study. The recruitment process was interrupted by the restrictions imposed during the COVID-19 pandemic. As such, we could only work with the sample size that we had when the pandemic started. Despite this, we were able to approach the same participants for a follow-up interview to which they all agreed, and by the end of the coding process, no new themes emerged suggesting that saturation had been reached. Furthermore, supporting evidence from a recent systematic review demonstrated that smaller sample sizes ranging from 9 to 17 interviews are sufficient to be considered as effective for qualitative research, because of the ability to reach saturation.^44^ The need to adapt this study amid the pandemic provided a unique opportunity to explore any potential changes in insights around barriers, facilitators, and key components of providing a sleep health program before and during a pandemic. In addition, despite collecting data from a convenience sample, participants were identified because of their role and influence within the workplace wellness division of their organizations and as such would play a pivotal role in the future of sleep health within their business. Nonetheless, the current study is explorative and provides preliminary insight from the WHP decision makers. Given this, our findings may provide the foundation from which future workplace sleep health programs can develop taken from those who have the impetus to drive such projects. Lastly, with regard to transferability, the results describe the perspectives of corporate executives and occupational medicine specialists in South Africa. Although some findings might only apply within a South African context, themes pertaining to the normalization of a demanding workplace culture and work-life balance within the corporate setting, may be transferrable to comparable settings worldwide.

### Implications/Recommendations

Valuable insights around provision of a sleep health component in WHP programs have emerged from this qualitative study. The insights on the barriers and facilitators specifically have implications for practice and policy. Our findings showed the need to expand sleep health awareness of corporate executives by educating them on the importance of sleep, particularly with respect to their work performance. By achieving this, sleep health promotion in its entirety can be further expanded to the remaining workforce. As such, effective communication to underscore the vital impact of good sleep health in relation to optimizing physical health, mental, and work performance is recommended. Ensuring that business leaders and decision makers within organizations understand the impact of sleep on health and work performance may lead to greater commitment in investing in sleep health management. For example, such communication to the decision makers within organizations can emanate by presenting evidence to indicate that corporate environments, which undermine sleep are harmful to the financial well-being of organizations. Furthermore, educating the key players of WHP development on the importance of sleep for health and cognitive functioning is necessary by emphasizing how poor sleep can have adverse short- and long-term effects.^12,45^ Therefore, the first step in contributing to better sleep outcomes for employees is for employers to recognize the importance of sleep and the adverse outcomes both for individuals and businesses stemming from insufficient sleep.

It is recommended that business leaders committed to supporting sleep health provide tailored worksite policies to maximize the potential for optimizing and maintaining sleep health. In this way, the business case for sleep health promotion can be emphasized within the corporate work environment and begin to shift the perception that lack of sleep equates to work dedication. Given this, a workplace culture that values sleep can be accomplished by implementing WHP programs focused on optimizing employee sleep health, educating employees about sleep health, and raising awareness thereof. The evident workplace culture observed in the present study suggests that those in managerial roles may still feel that substituting sleep time for work translates to being a better leader. To shift this demanding culture toward valuing and supporting sleep health, it has been proposed that employers encourage sleep health through various practices including the expansion of employee WHP budgets to incorporate sleep health.^35^ Moreover, it is paramount for business leaders to be aware of the sleep needs of employees. For this, the business case for sleep health needs to be clear and, based on our findings, may be realized through the education of the decision makers within organizations.

The WHP programs that include HRAs and fatigue risk assessments should provide a more comprehensive assessment dedicated to sleep health where sleep duration, quality, and sleep patterns are assessed. With sleep data collected during a sleep health screening, risk criteria can be calculated as per evidence-based sleep health guidelines. Having company sleep health data allows for the identification of employees who need further sleep health management, provides sleep health metrics to report on the sleep health status of the workforce, and allows for sleep health trends and management to be assessed over time. Currently, where sleep is concerned, occupational health practices cover workplace fatigue and typically focus on industries characterized by shift work, such as the railway industry, flight crews, medical professionals, and truck drivers.^46^ However, it is vital to recognize that daytime fatigue and sleep concerns in nonshift work settings may stem from different factors and thus require a different strategic approach when compared with shift workers. In addition, we propose that the occupational health sector be more active to promote sleep health especially because it has been acknowledged in a systematic review of the literature that the lack of knowledge related to sleep health represents a “gap” in the medical knowledge of occupational health doctors and nurses.^47^ Therefore, the comprehensive training and education of occupational health staff can enhance the sleep health risk screening process and sleep health management guidance accompanying it.

A sleep health program should acknowledge the importance of a healthy work-life balance. Contrasting the compulsory home confinement, which occurred during the pandemic, work flexibility, which includes hybrid working and being given the choice from where to work, may allow some employees to manage their day more effectively. This arrangement may help employees balance their work and personal lives better, resulting in reduced stress levels, increased productivity, and improved well-being.^48^ In the case of a pandemic, employers and supporting occupational healthcare professionals must be sensitive to individual employee needs or desires to alter work patterns to support work-life balance by considering family responsibilities and employee job demands. In this way, the barrier related to work-life imbalance may be overcome, encouraging employees to participate in a workplace sleep health program.

Finally, one needs to consider that this study addresses the corporate work setting. As such, the involvement of corporate executives in the participation of a sleep health program is highly encouraged and would demonstrate leadership support, which previously has been recognized as an important construct influencing employee participation in WHP programs.^49^ Having corporate executives participate in the sleep health program would send out a message that management understands the importance of sleep health and is prepared to devote considerable time and resources to identify and address sleep health issues.

## CONCLUSIONS

The perspectives from organizational leaders in the decision-making and planning process of WHP programs provided valuable insight into barriers, facilitators, and key components relating to workplace sleep health programs, before and during the COVID-19 pandemic. For corporate employees to participate in a sleep health program, organizations should re-evaluate their demanding workplace culture to embrace the importance of sleep health, which, in some instances, may require a cultural change in organizational thinking. The significance of sleep health awareness is unrefuted and needs to incorporate the importance of sleep health for well-being and work performance. Equally important is the enhancement of HRAs to include sleep-specific sections, which would identify those who need further sleep health management and provide baseline metrics for future program evaluation. As such, we recommend that stakeholders, such as employers, business leaders, and occupational health professionals who are involved in the development and implementation of WHPs, use this knowledge for the integration, development, and implementation of sleep health into future WHPs.
